# Multiplexed transcriptome analysis to detect *ALK, ROS1* and *RET* rearrangements in lung cancer

**DOI:** 10.1038/srep42259

**Published:** 2017-02-09

**Authors:** Toni-Maree Rogers, Gisela Mir Arnau, Georgina L. Ryland, Stephen Huang, Maruja E. Lira, Yvette Emmanuel, Omar D. Perez, Darryl Irwin, Andrew P. Fellowes, Stephen Q. Wong, Stephen B. Fox

**Affiliations:** 1 Peter MacCallum Cancer Centre, Melbourne, Victoria, Australia; 2 Oncology Research, Pfizer, San Diego, California, USA; 3 Agena Bioscience, Herston, QLD 4006, Australia; 4 Diagnostic Oncology, Pfizer, San Diego, California, USA

## Abstract

*ALK, ROS1* and *RET* gene fusions are important predictive biomarkers for tyrosine kinase inhibitors in lung cancer. Currently, the gold standard method for gene fusion detection is Fluorescence *In Situ* Hybridization (FISH) and while highly sensitive and specific, it is also labour intensive, subjective in analysis, and unable to screen a large numbers of gene fusions. Recent developments in high-throughput transcriptome-based methods may provide a suitable alternative to FISH as they are compatible with multiplexing and diagnostic workflows. However, the concordance between these different methods compared with FISH has not been evaluated. In this study we compared the results from three transcriptome-based platforms (Nanostring Elements, Agena LungFusion panel and ThermoFisher NGS fusion panel) to those obtained from *ALK, ROS1* and *RET* FISH on 51 clinical specimens. Overall agreement of results ranged from 86–96% depending on the platform used. While all platforms were highly sensitive, both the Agena panel and Thermo Fisher NGS fusion panel reported minor fusions that were not detectable by FISH. Our proof–of–principle study illustrates that transcriptome-based analyses are sensitive and robust methods for detecting actionable gene fusions in lung cancer and could provide a robust alternative to FISH testing in the diagnostic setting.

Lung cancer remains one of the major causes of cancer mortality in both men and women worldwide^1^. Genomic alterations identified in lung cancer have significant predictive value in the treatment of the disease including *EGFR* mutations found commonly in advanced non-small cell lung cancer (NSCLC) that are associated with a favourable response to tyrosine kinase inhibitors^2^,^3^.

More recently, structural genomic rearrangements involving the anaplastic lymphoma kinase (*ALK*) gene have been identified in NSCLCs. The most common rearrangement, occurring between *ALK* and echinoderm microtubule-associated protein-like 4 (*EML4*), is an inversion event resulting in the fusion of the 5′ end of *EML4* to the 3′ end of *ALK,* leading to constitutive kinase activity and malignant growth^4^. Multiple *EML4-ALK* variants have subsequently been described, all occurring in the same region of the *ALK* gene but involving different breakpoints within the *EML4* gene. Variants 1 and 3a/b account for the highest proportions, at 33% and 29% respectively^5^. Other fusion partners have also been described such as kinesin family member 5B (*KIF5B*), TRK-fused gene (*TFG*) and kinesin light chain 1 (*KLC1*)^5^,^6^,^7^,^8^. Subsequent studies have reported *ALK* rearrangements occurring in 3–5% of NSCLCs, where they are associated with younger patients who have a light or no smoking history^9^.

The development of kinase inhibitors such as crizotinib has led to a breakthrough in the treatment of NSCLC patients carrying *ALK* fusions, who gain significant survival benefit following treatment^10^. Crizotinib has recently shown therapeutic efficacy in NSCLC patients carrying other rearrangements and alterations, including those involving *ROS1* (c-ros oncogene 1), *RET* (rearranged during transfection), *MET* amplification and *MET* exon 14 deletion^11^,^12^,^13^,^14^. Detection of any of these rearrangements is therefore critical in the therapeutic management of patients with advanced disease.

Today, the gold standard method for gene fusion detection in lung cancer is Fluorescence *In Situ* Hybridization (FISH), however, this technique is expensive, labour intensive, requires expert pathology assessment, and is not amenable to multiplexing. Furthermore, unless multiple sections are taken at the same time, serial testing that occurs after a negative FISH result can waste time and valuable material. This is particularly pertinent in limited lung cancer clinical specimens where the starting material is often limited to a core needle biopsy. Other techniques such as immunohistochemistry (IHC) and reverse transcription-polymerase chain reaction (RT-PCR) exist, however are used with varying frequencies for *ALK, RET* and *ROS1* fusion detection depending on the laboratory’s acceptance of the analytical performance of the test^15^,^16^.

In recent times, the use of high-throughput transcriptome analysis and next-generation sequencing has provided major insights into the biological understanding of multiple cancers. These techniques not only provide the same analytical sensitivity and specificity as traditional diagnostic methods, but are also more adaptable to multiplexing and can therefore screen a larger array of clinically informative targets with minimal tissue sample. Subsequently, these methods can determine gene rearrangement presence as well as the specific variants.

In this work, we compared three different transcriptome–based methods for the detection of clinically relevant fusion genes using a collection of well-characterized formalin-fixed paraffin-embedded (FFPE) samples from NSCLC patients, as well as NSCLC cell lines. Although hundreds of new fusions have recently been reported^17^ and more will likely be discovered, in this paper we provide proof-of-principle for detection of current actionable fusions of high clinical relevance. We aimed to identify a multiplex method that was accurate, economical and straight-forward to implement in a pathology lab.

## Results

### FISH testing

Fifty-one NSCLC tumor samples were collected from eight different institutions as FFPE blocks. All samples were tested by FISH (Table 1) for *ALK, ROS1* and *RET* as part of routine diagnostic testing at the Peter MacCallum Cancer Centre Pathology Department.

Thirty-one NSCLC samples were FISH negative for *ALK, RET* and *ROS1*, 17 tested positive for *ALK*, two were *ROS1* positive and one sample was *RET* positive (Table 1). Two samples had inconclusive results with an atypical pattern observed by FISH and were thus classified as negative.

### Transcriptome analysis

#### Assessment of RNA yield and integrity

The tumor percentage of the surgically resected NSCLC samples ranged from 40% to 90% (as estimated by pathology review of an H&E stained section). After extraction, RNA yield varied from 300 ng to 34 ug of total RNA. The quality of the extracted RNA was assessed on a Bioanalyzer. As expected with FFPE-derived material, most of the samples had an RNA Integrity Number (RIN) below three and we thus used the percentage of RNA fragments greater than 300 bases as a measure of RNA integrity (Supplementary Figure 1). Our most severely degraded FFPE sample had only 7% of fragments above 300 bases, while the remaining samples ranged from 13% to 67%. In addition, the Agena LungFusion panel employed internal QC markers to assess sample yield and integrity, with the number of transcript copies of *GAPDH* and *EML4* measured to ensure enough cDNA was present for a reliable result.

#### Nanostring results

Nanostring uses a combination of capture and reporter probes to uniquely capture and tag a particular RNA molecule; only when the two probes hybridise close in the same transcript will a positive signal result. Three samples failed QC after normalization due to low input RNA (normalization factor >10) and were discarded and the results excluded from the percent agreement analyses. The number of counts for the four housekeeping genes in the assay ranged from 474 (100 ng RNA, sample SN09) to 139,323 counts (500 ng RNA, sample SN42). Not surprisingly, these two samples had 7% and 62% of molecules greater than 300 bases, respectively, reflecting the variability in clinical sample quantity and quality. The maximum number of counts for a single fusion variant was 1742 counts (SLC4A2ex4-ROS1) in the HCC-78 cell line, while in tissue, the maximum value was 680 counts (EML4_20-ALK_20) in sample SN08.

Although Nanostring provides software to assess data quality and to normalize values, there is no specific analysis pipeline for fusion detection, or a threshold at which a fusion is called positive. We therefore used the recommended analysis for gene expression established previously by Lira *et al*.^18^ and set the fusion threshold at 50 counts after normalization. Three probe sets (KIF5B_17-ALK_20, LRIG3_16-ROS1_35 and SDC4_4-ROS1_34) presented consistent high counts in all the samples and water controls, but were normalized by water subtraction (Supplementary Figure 2). However, probe KIF5B_23-RET_12 was removed due to non-specific binding to RNA.

Out of 48 FFPE samples remaining for analysis after normalization, only sample SN10 was negative using Nanostring but positive by FISH testing. In comparison, sample SN37 was assessed negative by FISH but was positive for an *ALK* fusion using Nanostring. The only *RET* positive case could not be tested because that particular fusion was not represented on the panel (CCDC6-RET_C1-R12). All other samples, including some with 100 ng of highly degraded RNA and variable amounts of tumor content, were in agreement (overall agreement of 96%) with 94% positive agreement and 97% negative agreement (Supplementary Table 1).

#### Agena results

Agena screens for known and *de novo ALK, RET* and *ROS1* translocations in a single reaction via four quantitative assays per gene (LungFusion v1.0). These assays firstly explore imbalanced expression between two exons in the same gene separated by the kinase domain with imbalanced expression indicative of an activating fusion product. When expression imbalance is detected, a confirmatory follow-up analysis is performed to characterise the fusion partner and breakpoint position at the exon level, using gene specific known fusion variant assays (AbA *ALK, RET* and *ROS1* Variant Panels) (Table 2).

In our cohort, after the first quantitative assay, two samples were discarded from further analysis due to poor cDNA quality and lack of fresh material to repeat the assay. The 3′–5′ imbalance ratio for estimation of over-expression ranged from 2:1 in a sample with only 25% RNA over 300 bases and 40% tumor content, to 71:1 for the H2228 cell line. Three samples gave positive *ROS1* fusions for both expression and fusion tests compared with FISH. However, two of these samples were marked as low level secondary *ROS1* results in the presence of a significant *ALK* fusion, where the *ALK* fusions were in concordance with FISH results (One false positive). Similar to the Nanostring platform, there were discordant results compared with FISH for samples SN37 and SN10 with Agena reporting a positive and negative result respectively. In contrast, Agena was the only platform to detect a FISH positive *RET* fusion in the most degraded sample, SN09. Agena also detected an unusual pattern in sample SN46, reported as atypical by FISH, suggesting the detection of a new fusion variant. Overall, the percent agreement was 94% showing 95% positive percent agreement and 93% negative percent agreement (Supplementary Table 1).

### ThermoFisher NGS

All samples produced more than 20,000 mapped reads (range 30,321–1,361,942). The most degraded sample (SN09) generated 534,718 mapped reads.

With standard threshold settings, all fusions were detected except the *RET* positive case SN09. However as mentioned above, this was the most degraded sample, also failing Nanostring QC as well as returning a borderline positive *RET* result in the Agena allele specific assay. The NGS fusion assay also detected three *ALK* positive fusions and one *ROS1* positive fusion not detected using any other platform and therefore potentially representing false positives. One of these samples, SN51, was scored as low cDNA quality based on Agena QC and gave one of the lowest numbers of fusion reads of any positive sample in the ThermoFisher NGS fusion assay (161 fusion reads, median of all positives samples, 3,297 fusion reads). Another discordant sample, SN55, had only 13% of its RNA above 300 bases on Bioanalyzer analysis, representing the second most degraded sample, and also possessing only 60% tumor content. Consistent with the Nanostring and Agena platforms, sample SN37 was also positive for an *ALK* fusion in the NGS fusion assay, suggesting the FISH result was likely a false negative. Moreover, across all three transcriptome platforms, SN10 was determined to be negative for *ALK* fusions, but was scored as positive by FISH. Significantly, this FISH case was noted as being difficult to enumerate and was ultimately reported as *ALK* fusion negative based on IHC (Supplementary Figure 3.). Overall, for the ThermoFisher NGS fusion platform, there was a positive percent agreement of 90% and a negative percent agreement of 84% (overall percent agreement of 86%) (Supplementary Table 1).

## Discussion

The recent discovery of druggable structural genomic alterations in lung and other cancers requires improved diagnostic methods that are capable of screening multiple fusions in a cost-effective, rapid and sensitive manner. In this paper we utilized three transcriptome-based platforms and compared results with the gold standard of FISH testing for the detection of *ALK, ROS1* and *RET* fusions in lung cancer.

Overall, the blinded evaluation of these three methods revealed all had a good concordance of results compared with FISH. However, due to the small cohort size, our statistical power for the analysis of *ROS1* and *RET* fusions was limited. Consequently, further assessment of these platforms in a larger cohort of samples would be warranted.

The RNA integrity was critical for the successful application of some of these platforms and while all samples successfully passed QC requirements for the ThermoFisher NGS fusion platform, there were a number of samples that were unable to be analyzed due to poor QC results from the Nanostring and Agena platforms. This suggests that upfront quality control measures that assess the RNA integrity of a sample will be integral in guiding the suitability of any assay. Pre-analytical variables such as fixation time and fixative will also be important factors to standardize if these platforms are to be used routinely.

Both FISH and Nanostring use unamplified nucleic acids and are therefore less sensitive to fixation effects, but at the same time are less prone to artefacts. This notion was supported by the Agena and ThermoFisher NGS fusion results, which showed a tendency to detect spurious or low level secondary fusions compared with Nanostring. However, because Nanostring employs no cDNA synthesis, PCR amplification, or enzymatic reaction step, its RNA input requirements are comparatively high. When RNA is of good quality, 100 ng is the recommended input requirement (or less with the latest XT chemistry, available subsequent to this work), but for FFPE samples we observed that higher sample inputs produced more reliable results. While FISH is technically challenging and not easy to multiplex, Nanostring in our experience, was extremely easy to operate with minimal hands-on time and can potentially multiplex over 200 targets in a single assay. Different strategies to identify fusions have additionally been developed using the Nanostring platform. For example, the nCounter Vantage Gene Fusion Panels from Nanostring combine probes that compare the ratio of gene expression upstream and downstream of a fusion junction (5′/3′ imbalance expression) and junction probes specific to the fusion breakpoint (toehold exchange technology).

Agena requires an initial cDNA synthesis step, followed by two rounds of specific amplification, labelling, and spectrometry. Although imbalance is a good method to discover new fusions within known partners, no additional fusion variants were observed. In several samples, low level secondary *ROS1* fusions were detected that were not identified by any other platform, including in samples with known *ALK* rearrangements. While these are potentially spurious, they may also represent real low-level fusion products, however further validation will be required to confirm their identity.

The ThermoFisher NGS Fusion panel has the advantage that it also provides sequence information and uses only 10 ng of total RNA, 10-fold less RNA than any of the other platforms tested. Other manufacturers have developed similar approaches including Archer™, QuantideX^®^, Clontech, and NuGEN. Moreover, with a single assay one can detect fusion breakpoints, imbalanced expression and DNA variants. However, this platform also demonstrated the most false positives, possibly due to sequencing artefacts and alignment errors, arguing that more refinements in the bioinformatic analysis are required. Cross-sample contamination is also a possibility that cannot be discounted, given the analytical sensitivity of this assay, and the open lab environment we used in this study. In a clinical setting, best practice contamination avoidance and detection measures should overcome any potential for contamination.

Interestingly, sample SN37 was assessed negative by FISH but all other platforms detected an *ALK* translocation, highly suggestive of a fusion product that was missed by FISH. In contrast, sample SN10 was determined to be *ALK* negative by all platforms but positive by FISH indicating this may be a false positive FISH result. Of note, this sample was reported to have a large amount of necrosis and an atypical FISH appearance, with many narrow breaks and non-specific staining. Ultimately this case was reported *ALK* fusion negative based on IHC. It is also notable for being the sole sample sourced from its pathology provider. These examples highlight how genomic methods can be more accurate and less subjective than FISH.

The utility of the platforms tested will be determined by the disease and type of material that can be obtained from the tissue, the sensitivity needed, and the clinical result required. For example, in a diagnostics lab, hybridization-based technologies seem to be more robust and more straight-forward for compliance (Elements is a general purpose reagent). In contrast, if the detection of a fusion is to be used as a biomarker for residual disease, then Agena or ThermoFisher NGS fusion panel might be more appropriate due to their higher sensitivity. The choice of which platform to deploy will also be dependent on what equipment and technical expertise is available at a testing site, with other factors, including the cost of testing and the volume of samples required for testing, determining the best platform to implement diagnostically. In a research setting, where the imperative to discover novel fusions rather than to detect those that are therapeutically actionable, sequencing will probably remain the preferred platform.

In conclusion, our findings provide a proof-of-principle that transcriptome-based analysis is a highly sensitive and effective means to identify multiple fusions, with comparable performance to FISH testing. While these platforms could become an alternative to FISH testing, further evaluation in a larger cohort of patients to assess their clinical accuracy will be needed.

## Methods

### Patient Samples and Cell Lines

We selected a total of 51 surgically resected NSCLC cases to be tested. To ensure the specific detection of the fusions across all platforms we included four cell lines (H3122 and H2228: *ALK* positive, HCC78: *ROS1* positive and RT-112: *ALK, ROS1* and *RET* negative) which had been previously tested by FISH to determine their *ALK, ROS1* and *RET* status (Supplementary Table 2). An FFPE cell pellet was created for H2228 to mimic the conditions of fixation used in clinical processing.

Ethics approval was obtained from the Human Research and Ethics Committees at the Peter MacCallum Cancer Centre (PMCC: 03/90) with all methods performed in accordance with the relevant ethical guidelines and regulations of the Australian National Health and Medical Research Council.

### Fluorescence *in situ* hybridization (FISH)

FISH was performed as previously described^19^. Two microlitres of the appropriate probe: *ALK* Break Apart Rearrangement Probe (Abbott Molecular, Des Plaines, IL, USA), *ROS1* Break Apart Rearrangement (gift from Translational Research Laboratory, Massachusetts General Hospital and Abbott Molecular, Des Plaines, IL, USA) and Zyto*Light*SPEC *RET* Dual Colour Break Apart Probe (ZytoVision GmbH, Bremerhaven, Germany) was placed onto the scribed tumor area.

Fifty tumor nuclei per case were assessed for each case using the following criteria:

For *ALK, ROS1* and *RET* FISH, a case was considered positive (rearranged) if 15% or greater of tumor cell nuclei were rearranged.

For *ALK* FISH, cells were considered positive (rearranged) if there was a split two or more signal widths apart between the orange and green signals or there was a single orange signal (3′ end) without a corresponding green signal (5′ end) in conjunction with a fused and/or split signals. For *ROS1* FISH, cells were considered positive (rearranged) if there was a split two or more signal widths apart between the orange and green signals. A case was considered atypical if there was a single orange signal (3′ end) without a corresponding green signal (5′ end) in conjunction with a fused and/or split signals, or if there was a single green signal (5′ end) without a corresponding orange signal (3′ end) in conjunction to a fused and/or split signals. For *RET* FISH, cells were considered positive (rearranged) if there was a split two or more signal widths apart between the red and green signals.

### RNA Extraction

For the FFPE samples, total RNA was isolated from 2 to 10 sections (7 μm thickness) using the QiagenRNeasy^®^ FFPE kit (Qiagen, Hilden, Germany). For the cell lines total RNA was isolated using the QiagenRNeasy^®^ Mini kit (Qiagen, Hilden, Germany). The manufacturer’s protocol was followed with the addition of a DNase treatment step (Ambion TURBO DNA-free™ kit, Life Technologies, Grand Island, NY, USA). RNA concentration was assessed using Nanodrop2000 Spectrophotometer (Thermo Scientific, Grand Island, NY, USA) and RNA quality was assessed using the Agilent 2100 Bioanalyzer (Agilent Technologies, Santa Clara, CA, USA).

### Nanostring Fusion Assay

Custom probe sets were designed by Nanostring Technologies (Nanostring Technologies, Seattle, WA, USA). We used nCounter Elements^TM^ General Purpose Reagents and probes synthesized by IDT (Integrated DNA Technologies Pte Ltd, Singapore). With the Elements chemistry, two sets of probes are designed per fusion: probe A hybridizes to the 5′ end of the fused transcript and to the Elements Reporter Tag, and probe B hybridizes to the 3′ end of the transcript and to the Elements biotin-labelled Universal Capture Tag. Only transcripts binding two probes will be captured and reported. Our design consisted of 29 probe sets based on Lira *et al*.^18^,^20^ targeting several known *ALK, RET* and *ROS1* fusion forms and four housekeeping genes for content normalization (*GADPH, GUSB, OAZ1* and *POLR2A*).

The Nanostring Elements protocol for gene expression was followed. Briefly, 100–500 ng of total RNA was hybridized to nCounter Elements and IDT probe sets at 67 °C for 20 hours. After hybridization, samples were immobilized and washed using an automated nCounter Prep Station (Nanostring Technologies, Seattle, WA, USA), then imaged on a Digital Analyser (Nanostring Technologies, Seattle, WA, USA) programmed at 550 fields of view.

Analysis was done using the nSolver 2.6 (Nanostring Technologies, Seattle, WA, USA) following Nanostring’s recommendations for gene expression. For technical normalization we used the following settings: negative control probes, subtract max; positive control probes, geomean. Blank samples (water only) were then subtracted from all samples to eliminate unspecific hybridization (mean plus 2 SD), and resultant negative values were brought to zero. Counts were further normalized with the four housekeeping genes to correct for input RNA using the geomean as scaling factor. Values above 50 counts after normalization were considered positive, all other values negative.

### Agena Lung Fusion Panel

Two hundred to five hundred nanograms of total RNA was converted to cDNA using an Invitrogen SuperScript^®^ VILO cDNA synthesis kit (Life Technologies, Carlsbad, CA, USA) and random hexamer priming according to manufacturer’s recommendations. cDNA was processed, analyzed and reported by Agena Bioscience Inc. Applications Development Division, Herston, QLD, Australia. Multiplex screening of the samples was performed using the MassARRAY LungFusion v1.0 Panel (Agena Bioscience, San Diego, CA, USA) followed by a LungFusion Assay by Agena (AbA) *ALK, RET* and *ROS1* Variant Panels (Agena Bioscience, San Diego, CA, USA) for further characterization of fusion positive samples.

The LungFusion v1.0 Panel screens for the presence of known and *de novo* fusions in *ALK, RET* and *ROS1* genes by enumerating transcript copy number in pre- and post-kinase domains and inferring expressed chimeric oncogene via differential expression (Table 2). Briefly, 5–10 ng of cDNA per sample was PCR amplified in the presence of a known copy number competitive template standard within a single multiplex reaction with primers designed immediately 3′ of the promoter and the kinase domain. Unincorporated dNTPs were inactivated and then a single base extension reaction was performed generating a unique mass product for each site. The reaction was conditioned with ion exchange resin and 5–15 nl spotted onto a 384-padSpectroCHIP (Agena Bioscience, San Diego, CA, USA) using the Nanodispenser RS-1000 (Agena Bioscience, San Diego, CA, USA). Masses of the analytes were obtained by matrix-assisted laser desorption/ionization time-of-flight (MALDI-TOF) mass spectroscopy on the MassARRAY 4 Analyser (Agena Bioscience, San Diego, CA, USA). Analysis was performed by the Typer 4 software (Agena Bioscience, San Diego, CA, USA) and R package version 2.9.1 (The R Foundation for Statistical Computing, Vienna, Austria) to assess pre- and post-kinase domain expression.

Samples with an increased post-kinase domain transcription were selected for further analysis to determine the specific known fusion with the *ALK, RET* or *ROS1* Variant specific Assay by Agena (AbA) Panels (Supplementary Table 3).

### ThermoFisher NGS fusion panel

Total RNA was analyzed by EA Genomic Services (Q2 Solutions, Durham, NC, USA). The ThermoFisher NGS Fusion Assay uses AmpliSeq assay technology and Ion Torrent sequencing (ThermoFisher Scientific Waltham, MA USA) for the targeted sequencing of known and *de novo* fusion junctions for *ALK, RET, ROS1* and *NTRK1*. Reverse transcription is followed by multiplex PCR using a single primer pool. Briefly, libraries were prepared from 10 ng of total RNA via reverse transcription multiplex PCR, and barcoded sequencing adapters ligated to the resulting amplicons. Libraries were pooled, amplified in emulsion PCR and sequenced on the Ion Torrent PGM instrument (ThermoFisher Scientific Waltham, MA USA). Eight samples were batched on a single 318 PGM chip to give >150,000 reads per sample. Raw data was transferred to the Torrent Suite software server (ThermoFisher Scientific Waltham, MA USA) for alignment and to Ion Reporter software v4.4 (ThermoFisher Scientific Waltham, MA USA) for variant calling. After sequencing, reads are aligned and for samples with more than 20,000 mapped reads, fusions were detected when the sequence of one gene was found relocated to that of another gene at the breakpoint (Table 2). A fusion was called when more than 40 fusion reads supported the breakpoint. Fusions were also inferred qualitatively from differential expression data, where expression of the 5′ and 3′ ends of a transcript were significantly imbalanced in fusion samples compared with normal samples. Assay performance was controlled using the expression of five stable control genes built into the panel for quality checking purposes.

## Additional Information

**How to cite this article**: Rogers, T.-M. *et al*. Multiplexed transcriptome analysis to detect *ALK, ROS1* and *RET* rearrangements in lung cancer. *Sci. Rep.* **7**, 42259; doi: 10.1038/srep42259 (2017).

**Publisher's note:** Springer Nature remains neutral with regard to jurisdictional claims in published maps and institutional affiliations.

## Supplementary Material

Supplementary Material

## Figures and Tables

**Table 1:** Summary of fusion detection results.

Sample Number (SN)	FISH			Nanostring			Agena LungFUSION panel			ThermoFisher NGS			Comment
	*ALK*	*RET*	*ROS1*	*ALK*	*RET*	*ROS1*	*ALK*	*RET*	*ROS1*	*ALK*	*RET*	*ROS1*	
01	P	nk	nk	P	N	N	P	N	N	P	N	N	
02	N	N	N	N	N	N	N	N	N	N	N	N	
03	N	N	N	N	N	N	N	N	N	N	N	N	
04	P	nk	nk	P	N	N	P	N	N	P	N	N	
05	P	nk	nk	P	N	N	P	N	N	P	N	N	
06	P	nk	nk	P	N	N	P	N	N	P	N	N	
07	P	nk	nk	P	N	N	P	N	N	P	N	N	
08	P	nk	nk	P	N	N	P	N	N	P	N	N	
09	nk	P	nk	F	F	F	N	P	N	N	**N**	N	Low *RET* Agena
10	P	N	N	**N**	N	N	**N**	N	N	**N**	N	N	FISH +ve cells 1–50, −ve cells 51–100, IHC −ve, some narrow breaks, non-specific staining
11	N	N	N	N	N	N	F	F	F	N	N	N	Normal in Agena but below threshold
13	P	nk	nk	P	N	N	P	N	N	P	N	N	
14	N	N	N	N	N	N	N	N	N	N	N	N	
15	N	N	N	N	N	N	N	N	N	N	N	N	
16	N	N	N	N	N	N	N	N	N	N	N	N	
17	N	N	N	N	N	N	N	N	N	N	N	N	
18	P	nk	nk	P	N	N	P	N	N	P	N	N	
19	N	N	N	N	N	N	N	N	N	N	N	N	
20	N	N	N	N	N	N	N	N	N	N	N	N	
21	N	N	N	N	N	N	N	N	N	N	N	N	
22	N	N	N	N	N	N	N	N	N	N	N	N	
23	N	N	N	N	N	N	N	N	N	N	N	N	
24	P	nk	nk	P	N	N	P	N	N	P	N	N	
25	N	N	N	N	N	N	N	N	N	N	N	N	
26	P	nk	nk	P	N	N	P	N	N	P	N	N	
27	nk	N*	N*	N	N	N	N	N	N	N	N	N	FISH atypical for *RET* and *ROS1*
29	P	nk	nk	F	F	F	P	N	N	P	N	N	Failed Nanostring normalization, although *ALK* fusion was observed
30	N	N	N	N	N	N	N	N	N	N	N	**P**	
32	nk	nk	P	F	F	F	N	N	P	N	N	P	
33	P	N	N	P	N	N	P	N	N	P	N	N	Low *ROS1* Agena detected in variant specific
34	N	N	N	N	N	N	N	N	N	N	N	N	
35	N	N	N	N	N	N	N	N	N	N	N	N	
36	N	N	N	N	N	N	N	N	N	N	N	N	
37	N	N	N	**P**	N	N	**P**	N	N	**P**	N	N	
38	nk	nk	P	N	N	P	N	N	P	N	N	P	
39	N	N	N	N	N	N	N	N	**P**	N	N	N	Low *ROS1* Agena detected in variant specific
40	N	N	N	N	N	N	N	N	N	N	N	N	
41	N	N	N	N	N	N	N	N	N	N	N	N	
42	P	nk	nk	P	N	N	P	N	N	P	N	N	
43	P	nk	nk	P	N	N	P	N	N	P	N	N	
44	N	N	N	N	N	N	N	N	N	N	N	N	
45	P	nk	nk	P	N	N	P	N	N	P	N	N	
46	nk	nk	N*	N	N	N	N	N	N	N	N	N	FISH atypical for *ROS1*
47	N	N	N	N	N	N	N	N	N	N	N	N	
48	P	nk	nk	P	N	N	P	N	N	P	N	N	Agena–High *ALK* + Low *ROS1* detected, *ROS1* not confirmed by variant analysis–reportable sample result = *ALK* Fusion Positive
49	N	N	N	N	N	N	N	N	N	N	N	N	
50	N	N	N	N	N	N	N	N	N	**P**	N	N	
51	N	N	N	N	N	N	N	N	N	**P**	N	N	
52	N	N	N	N	N	N	F	F	F	N	N	N	Normal in Agena but below threshold
53	N	N	N	N	N	N	N	N	N	N	N	N	
55	N	N	N	N	N	N	N	N	N	**P**	N	N	

N = Negative, P = Positive, nk = not done or not known, F = Failed test result, Atypical by FISH *, Discordant cases compared to FISH are highlighted in bold font.

**Table 2:** Technical basis and performance characteristics of each platform.

Platform	Nanostring	Agena LungFUSION panel	ThermoFisher NGS
Panel	Custom	Imbalance 5′-3′ + LungFUSION panel v1.0	Ion AmpliSeq™ Colon and Lung Cancer Research Panel v2
Principle	Sequence specific hybridization	Reverse Transcription, PCR, Primer extension, MALDI-TOF	Targeted next generation sequencing on the fusion junction. Reverse Transcription occurs in the same tube.
Tolerates FFPE?	Y	Y	Y
Input requirements	100–300ng RNA	100–500ng RNA	10 ng RNA
cDNA synthesis?	N	Y	Y
Genes in the panel	*ALK, RET, ROS1*	*ALK, RET, ROS1*	*ALK, RET, ROS1, NTRK1*
Customizable?	Y	Y	Y
5′-3′ expression?	N (possible)	Y (*ALK, ROS1 and RET*)	Y (*ALK*)
Analysis software?	Y (nSolver)	Y (MassArray Typer 4)	Y
Result report?	N	Y (LungFusion Report)	Y (Ion Reporter Software)
HK standards	*GADPH, GUSB, OAZ1* and *POLR2A*	*GAPDH and EML4*	

Y = Yes, N = No.
