# Supplementary material for: Multiplexed transcriptome analysis to detect ALK, ROS1 and RET rearrangements in lung cancer
Source: Sci Rep. 2017 Feb 9;7:42259. doi: 10.1038/srep42259 (PMC5299839; doi:10.1038/srep42259)
Supplement: Supplementary Material [file srep42259-s1.pdf]

Supplementary Material

Multiplexed transcriptome analysis to detect *ALK, ROS1* and *RET* rearrangements in lung cancer

Toni-Maree Rogers, Gisela Mir Arnau, Georgina L. Ryland, Stephen Huang, Maruja E. Lira, Yvette Emmanuel, Omar D. Perez, Darryl Irwin, Andrew P. Fellowes, Stephen Q. Wong, Stephen B. Fox

Supplementary Table 1. Percent agreement metrics

Positive percent agreement:PPA	Negative percent agreement: NPA	Overall percent agreement: OPA

FISH						
	ALK	Positive	Negative	PPA	NPA	OPA
**Nanostring**	**Positive**	**15**	**1**	**94%**	**97%**	**96%**
	Negative	1	31			
	Total	16	32			
	RET	Positive	Negative	PPA	NPA	OPA
**Nanostring**	Positive	0	0	N/A	100%	100%
	Negative	0	48			
	Total	0	48			
	ROS	Positive	Negative	PPA	NPA	OPA
**Nanostring**	Positive	1	0	100%	100%	100%
	Negative	0	47			
	Total	1	47			
	All fusions	Positive	Negative	PPA	NPA	OPA
**Nanostring**	Positive	16	1	94%	97%	96%
	Negative	1	30			
	Total	17	31			

FISH						
	ALK	Positive	Negative	PPA	NPA	OPA
**Agena**	**Positive**	**16**	**1**	**94%**	**97%**	**96%**
	Negative	1	31			
	Total	17	32			
	RET	Positive	Negative	PPA	NPA	OPA
**Agena**	Positive	1	0	100%	100%	100%
	Negative	0	48			
	Total	1	48			
	ROS	Positive	Negative	PPA	NPA	OPA
**Agena**	Positive	2	1	100%	94%	98%
	Negative	0	46			
	Total	2	47			
	All fusions	Positive	Negative	PPA	NPA	OPA
**Agena**	Positive	19	2	95%	93%	94%
	Negative	1	27			
	Total	20	29			

FISH						
	ALK	Positive	Negative	PPA	NPA	OPA
**ThermoFisher NGS**	**Positive**	**16**	**4**	**94%**	**88%**	**90%**
	Negative	1	30			
	Total	17	34			
	RET	Positive	Negative	PPA	NPA	OPA
**ThermoFisher NGS**	Positive	0	1	N/A	98%	98%
	Negative	0	50			
	Total	0	51			
	ROS	Positive	Negative	PPA	NPA	OPA
**ThermoFisher NGS**	Positive	2	1	100%	98%	98%
	Negative	0	48			
	Total	2	49			
	All fusions	Positive	Negative	PPA	NPA	OPA
**ThermoFisher NGS**	Positive	18	5	90%	84%	86%
	Negative	2	26			
	Total	20	31			

Supplementary Table 2. Description of positive control cell lines.

Cell line name	Specific fusion
H2228 (FFPE)	ALK/EML4 v. 3a & 3b
RT-122	FGFR3-TACC3
H3122	ALK/EML4 v. 1
HCC-78	SLC34A2-ROS1

Supplementary Table 3. Specific fusion detected using the ALK, RET or ROS1 Variant specific Assay by Agena (AbA) Panels

Gene	Variant
EML4;ALK	Ee13;Ae20
	Ee20;Ae20
	Ee6a/b;Ae20
	Ee14;ins11del49Ae20
	Ee2;Ae20 & Ee2;ins117Ae20
	Ee13;ins69Ae20
	Ee14;del12Ae20
	Ee15del19;del20Ae20
	Ee18;Ae20
KIF;ALK	Ke17;Ae20
	Ke24;Ae20
KIF5B;RET	Ke15;Re12
	Ke16;Re12
	Ke22;Re12
	Ke23;Re12
	Ke24;Re11
CCDC6;RET	Ce1;Re12
FIG(GOPC);ROS1	Fe7;Re35
	Fe3;Re36
TPM3;ROS1	Te8;Re35
SDCC4;ROS1	Se2;Re32
	Se4;Re32
	Se4;Re34
SLC34A2;ROS1	Se12;Re32
	Se12;Re34
	Se4;Re32
	Se4;Re34
CD74;ROS1	Ce6;Re32
	Ce6;Re34
EZR;ROS1	Ee10;Re34
LRIG3;ROS1	Le16;Re35

* **Ee13;Ae20** = **E**LM4 **e**xon **13** fused with **A**LK **e**xon **20**

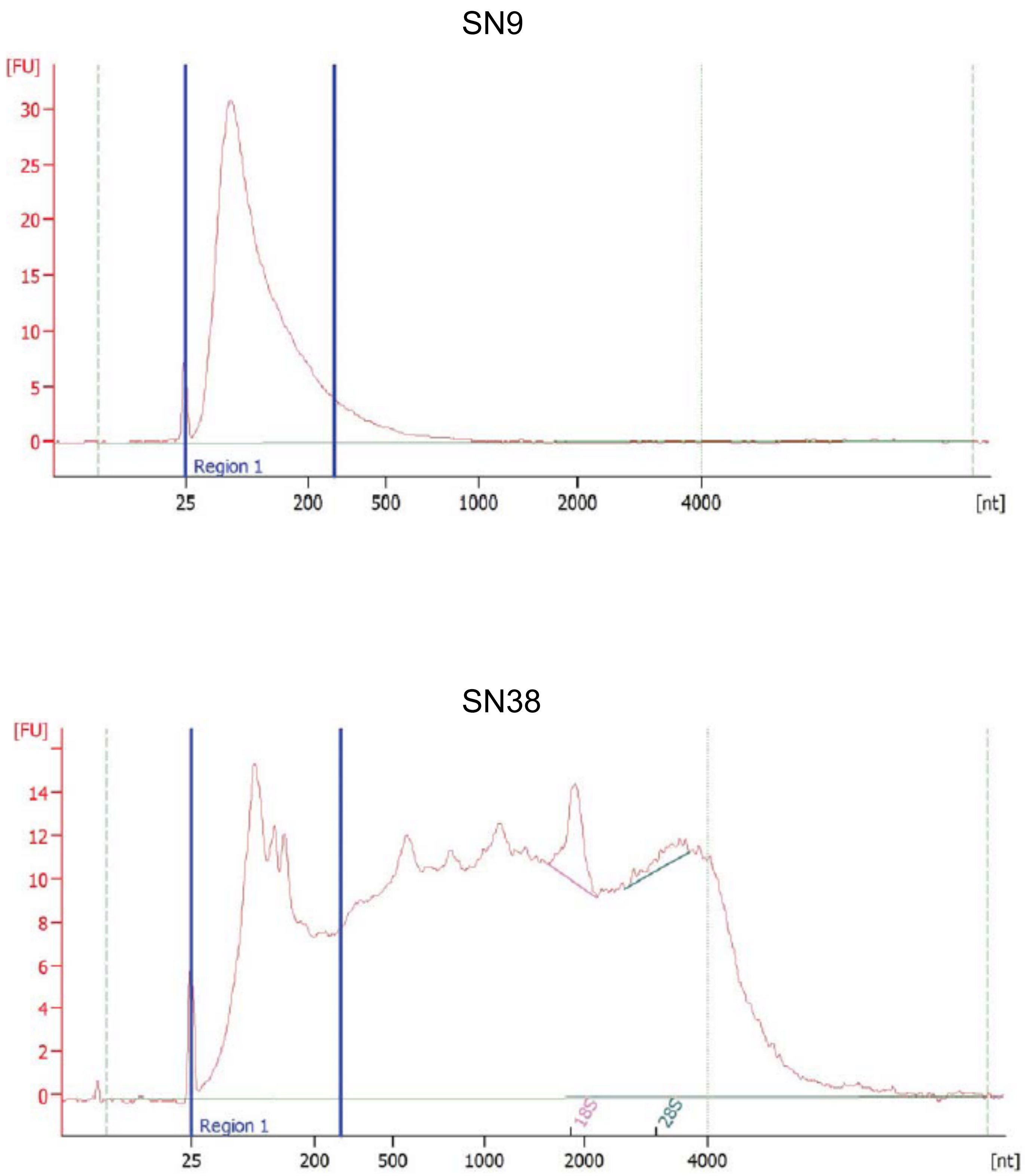

Supplementary Figure 1. Representative Bioanalyser images for two clinical NCSLC samples, i.e. SN9 representing a poor quality RNA sample and SN38 representing a good quality sample.

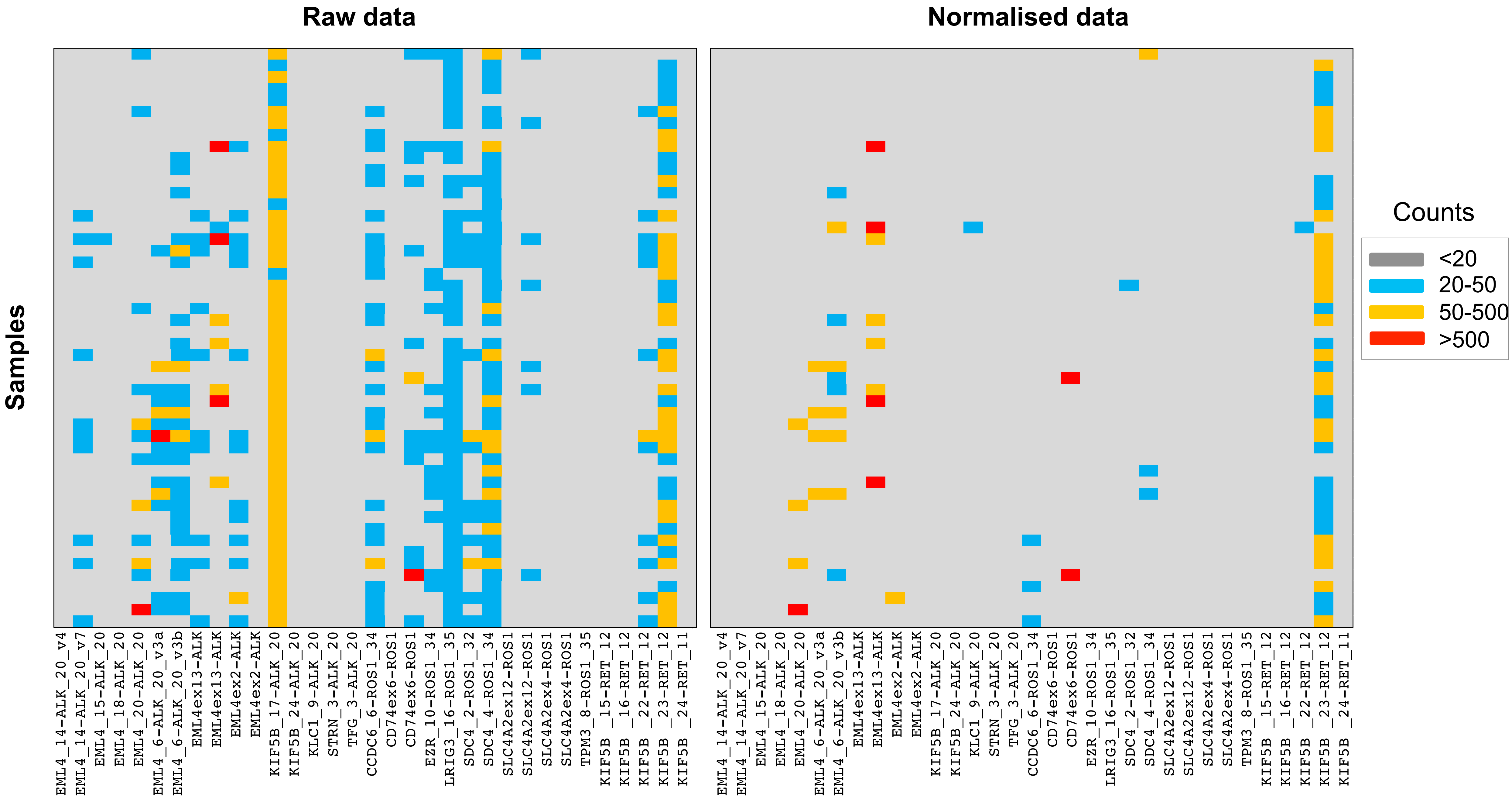

Supplementary Figure 2. Raw vs. normalised values in the Nanostring analysis. After normalisation, counts above 50 were considered positive.

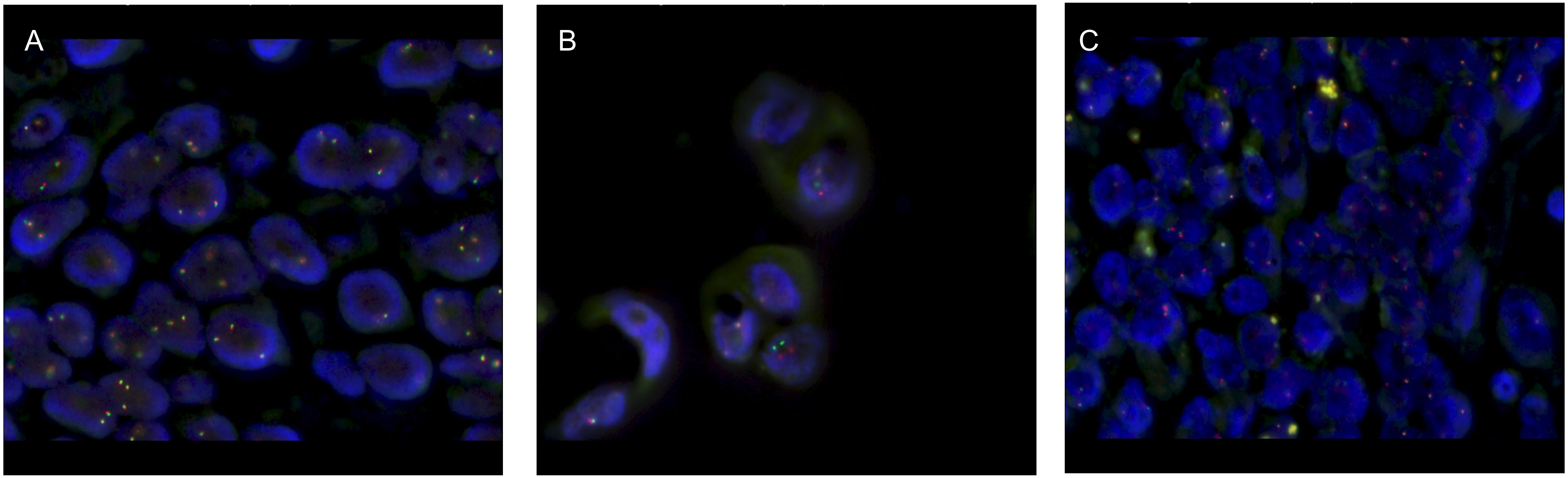

Supplementary Figure 3. ***ALK*** **signal patterns observed by FISH.** (A) A non-*ALK*-rearranged tumor. (B) An *ALK*-rearranged tumor with a split (greater than two signal widths apart). (C) Sample SN10, An atypical *ALK* FISH result with many narrow breaks and non-specific staining.
